# Free Flap Reconstruction of Traumatic Skin Defects of the Entire Hand Dorsum

**DOI:** 10.3390/jcm14041308

**Published:** 2025-02-16

**Authors:** Soyeon Jung, Seungjun Lee, Seokchan Eun

**Affiliations:** 1 Department of Plastic and Reconstructive Surgery, Hallym University College of Medicine, Hallym University Dongtan Sacred Heart Hospital, Hwaseong 18450, Republic of Korea; ps.soyeon.jung@gmail.com; 2 Department of Plastic and Reconstructive Surgery, Seoul National University College of Medicine, Seoul National University Bundang Hospital, Seongnam 13620, Republic of Korea; winters88@naver.com

**Keywords:** hand defect, hand reconstruction, free flap

## Abstract

**Background/Objectives**: The reconstruction of hand defects, especially involving the dorsal region of the hand, has remained a challenge for surgeons because of its anatomical features and complex functions. The goal of reconstruction should include functional restoration as well as being esthetically pleasing. The flap transfer reconstruction strategy is essential for satisfying these requirements. **Methods**: Free flaps were used to cover traumatic defects of the hand dorsum in eleven patients from 2016 to 2022. Eight males and three females with a mean age of 41 years were enrolled. The size of the flaps ranged from 6 × 5 cm to 20 × 9 cm, and the selected flaps included five anterolateral thigh flaps, three lateral arm flaps, and three superficial circumflex iliac artery flaps. **Results**: All flaps survived, with one case of partial necrosis. One patient experienced joint stiffness during recovery. The donor sites were closed primarily, and there was no need for skin grafting. Secondary debulking or thinning was also not required. The majority of cases recovered excellent function of the hand (mean Q_DASH: 2.5) with satisfactory esthetic outcomes. The postoperative observations were followed by more than six months. **Conclusions**: Small defects can be treated using local, pedicled, and island-type flaps. However, larger defects involving the exposure of tendons, nerves, and other critical structures commonly require free tissue transfers. The flap reconstruction for hand dorsum in the study is feasible to produce acceptable outcomes in large sized defects.

## 1. Introduction

Large soft tissue defects of the hand dorsum are infrequent, but reconstruction can be difficult depending on the mode and type of injury [1]. Unlike the palm area, the dorsal part of the hand is mobile, stretchable, and prone to injury under shearing forces. Subsequently, the tendons and bones are exposed, and appropriate soft tissue resurfacing is necessary. Various options for reconstruction are available to treat dorsal hand defects [2]. The distal pedicled radial forearm flap or the posterior interosseous flap can be used for small- to medium-sized defects [3,4]. However, large defects with the exposure of critical structures such as bones, tendons, and joints require free flap transfer to achieve successful resurfacing. Thus, free flap transfer is beneficial for providing sufficient tissue with color and texture matching, which enables the satisfactory contour of the hand to be achieved. However, some free flaps require the sacrifice of artery and the risk of poor circulation, microsurgical procedures, and the risk of failure [5,6]. To our knowledge, very few studies have been published on dorsal hand reconstruction to date; thus, the aim of this study was to present and share our experiences of dorsal hand reconstruction using free flap transfer.

## 2. Materials and Methods

Between 2016 and 2022, free flap transfers for the reconstruction of traumatic defects of the hand dorsum were carried out on patients by an experienced senior surgeon in a single center. The study was conducted retrospectively (Level of evidence: Level IV), and the patients were enrolled from the senior surgeon’s case data. Their ages were ranged from 28 to 72 and the gender was six males and five females, respectively. They suffered from diverse types of traumas, such as traffic or heavy machinery accidents.

All the reconstructive surgeries were performed under general anesthesia and supine-positioned. The tourniquet was applied only when the lateral arm flap was elevated. The following immobilization, recovery was dependent on the stability of the transferred flap from two to three weeks.

Adequate debridement was performed to remove non-viable tissue; subsequently, adjacent recipient artery and veins were dissected and prepared. The flap dimensions were designed to be large enough to cover the defect, incorporating as many veins as possible. Each flap was raised above the underlying muscle fascia using the thinned flap technique, and a pedicle of sufficient length was obtained. All donor sites were closed primarily with no skin grafting. The arterial anastomosis of the dorsal branch of the radial artery continued in an end-to-side fashion near the anatomical snuffbox. A silicone drain was placed under the flap, and sutures were placed between the flap and the surrounding skin. Ancillary procedures for the debulking or thinning of the flap were not required. Post-operative flap monitoring was performed in accordance with the protocol of the institution. The flap monitoring protocol included assessments of flap temperature, color, capillary refill, and checking the vascular patency using acoustic Doppler (Bidop7, Hadeco, Inc., Kawasaki, Japan). The frequency of monitoring was conducted every two hours until the third day after surgery. Post-operative outcomes were assessed in esthetics and functions. The assessments were performed by a hand specialist in the department who did not take part in the operation or therapy. All the patients were evaluated with the Quick Disabilities of the Arm, Shoulder, and Hand (Q-DASH) score at 12 months following surgery [7]. A survey based on a subjective questionnaire for the esthetical satisfaction was also conducted and graded on a five-point Likert scale (Unsatisfactory, Poor, Fair, Good, and Excellent) [8].

## 3. Results

The flap included four anterolateral thigh flaps, three lateral arm flaps, and four superficial circumflex iliac artery flaps with sizes ranging from 6 × 5 cm^2^ to 20 × 9 cm^2^ (Table 1). The ratio of males to females was six (54%) to five (46%). The average age of the patients was 44.6 years at the time of surgery. Ten flap transfers were successful with no adverse events, and only one suffered from partial necrosis. One patient experienced metacarpophalangeal joint stiffness following surgery. The texture, color, and thickness of the transferred flaps were satisfactory and provided excellent coverage of bones, joints, and tendons. The restoration of sensation was acceptable, with protective sensation regained within six to twelve months. The post-operative observations were conducted at a minimum of six months to three years. All eleven patients were satisfied with the results and gave a score of good to excellent for esthetics and function. The mean that the Q-DASH score was 2.5 (range: 0–4.2) and esthetical satisfaction was shown to be three patients in Fair, five in Good, and three in Excellent, respectively.

### 3.1. Case Series

#### 3.1.1. Patient 1

**Figure 1:**
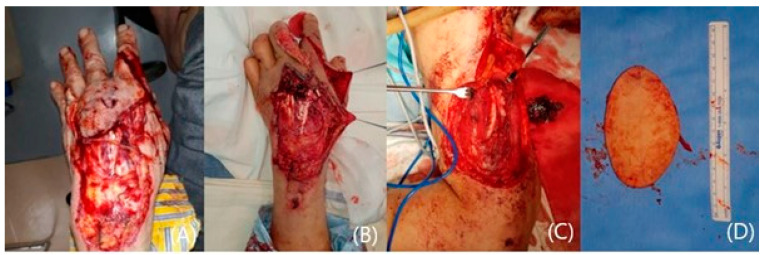
A 72-year-old male showing the extensive defect on his hand dorsum (**A**). Note the defect exposing the tendons (**B**). A lateral arm flap elevation and the harvested flap (**C**,**D**).

**Figure 2:**
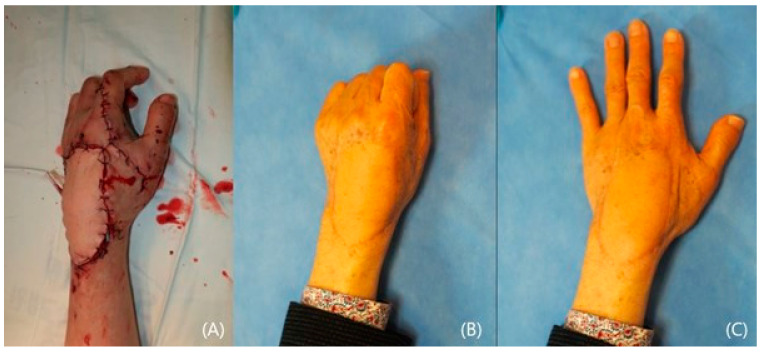
The transferred and sutured flap (**A**). Postoperatively 2-years photographs with motion (**B**,**C**). Note the acceptable contour and the motion.

A 72-year-old male was involved in a traffic accident and visited an emergency care center complaining of large soft tissue damage with tendon exposure on his left dorsal hand (Figure 1A,B). Following adequate debridement, a lateral arm free flap with dimensions of 10 × 6 cm^2^ on the same side was designed and elevated (Figure 1C). The raised flap was transferred to the defect, and the posterior radial collateral artery was connected to the dorsal branch of the radial artery (Figure 1D). One vena comitans was anastomosed to the cephalic vein. The donor site was closed primarily. The transferred flap survived well, and the long-term result over 2 years was satisfactory (Figure 2A). The contour was excellent, showing no limitations in terms of wrist and hand motions (Figure 2B,C).

#### 3.1.2. Patient 2

**Figure 3:**
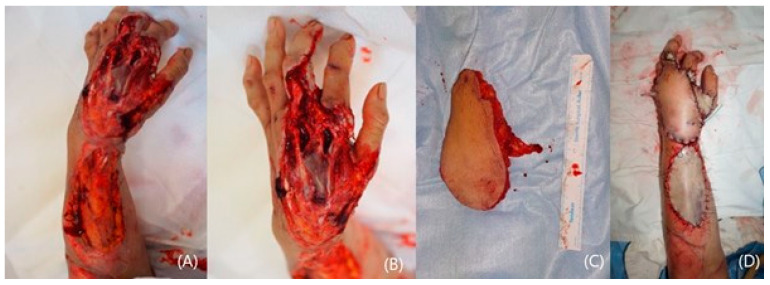
A 57-year-old female with extensive traumatic defect exposing critical structures (**A**,**B**). A raised ALT flap and the completion of flap transfer (**C**,**D**). A full-thickness skin grafting on her forearm region (**D**).

**Figure 4:**
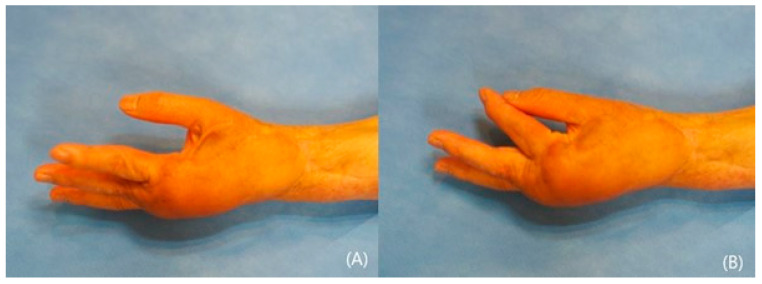
Post-operative pictures after 3 years (**A**,**B**). Note the acceptable contour and pinching.

A 57-year-old female acquired a traumatic defect to her left hand dorsum from a car accident. The dorsal defect extended to the wrist and forearm, exposing ruptured tendons and fractured phalange on her hand. The soft tissue on her wrist and forearm mostly remained (Figure 3A,B). An anterolateral thigh flap (ALT) was planned to resurface the dorsal hand. The size of the flap was 12 × 8 cm^2^ and was able to cover the hand and wrist (Figure 3C). The full-thickness skin was harvested from the groin and grafted onto the forearm (Figure 3D). Proximal interphalangeal joint fusion was required after eight months of observations. The result at 3 years was satisfactory, showing acceptable contour and pinching (Figure 4A,B).

#### 3.1.3. Patient 3

**Figure 5:**
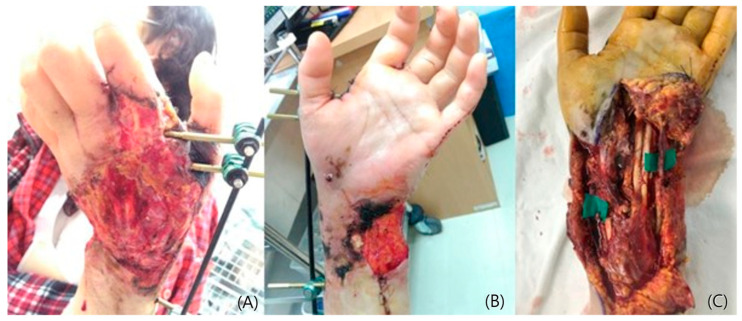
A 65-year-old female presenting multiple fractures and soft tissue defects on her left hand and wrist (**A**,**B**). A photograph showing the injured structures during wide debridement (**C**).

**Figure 6:**
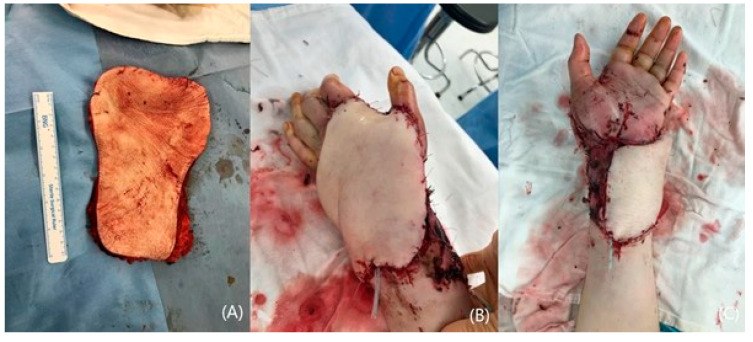
A photograph of elevated SCIP flap (**A**). The transferred flap and completion of inset (**B**,**C**).

**Figure 7:**
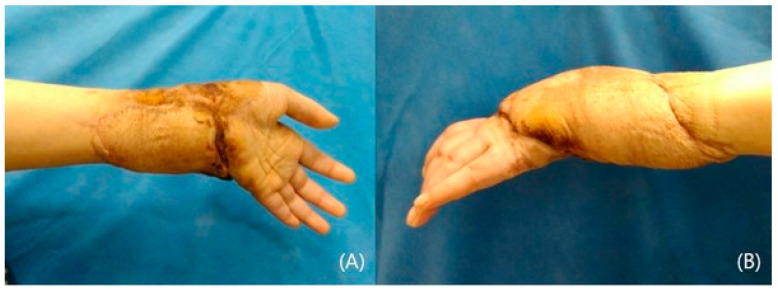
Post-operative photographs after 6 months (**A**,**B**).

A 65 year-old female with extensive damage to her left hand and wrist was referred to the hospital. Distal radius and scaphoid fractures accompanied the injury (Figure 5A). The distal circulation was not sufficient due to the rupture of the radial artery and clotting in the ulnar artery (Figure 5B). A free flap reconstruction was planned following debridement for necrotic tissue and the preservation of the tendon structure (Figure 5C). A superficial circumflex iliac artery perforator flap (SCIP) was designed and elevated (Figure 6A). The transferred flap was sutured to the recipient site and arterial anastomosis to the repaired radial artery was continued (Figure 6B,C). The donor site was closed directly. The post-operative observation period was uneventful, and the flap settled well (Figure 7A,B).

## 4. Discussion

The hand is one of the most functional parts of the human body. Thus, damage to the hand affects the quality of daily life when considering its esthetics and functions. Industrial development has led to an increase in accidents and other trauma to the hands [9,10]. In particular, the dorsal skin and soft tissue of the hand are vulnerable to injuries that cause traumatic defects. Moreover, the thinness of the skin means that tendons, vessels, and bones are prone to exposure from diverse injuries [10,11]. Early wound closure or reconstruction is critical to treat extensive dorsal injuries regardless of their size. Delayed reconstruction increases the likelihood of secondary procedures, longer hospital stays, and complications. Although there are many types of flaps that can be used in reconstruction, most are unreliable or unsatisfactory for dorsal hand restoration. The surgeon’s experience, technical developments, and hospital facilities are factors affecting the selection of a flap. When considering the features of the dorsal side of the hand, thin and stretchable tissue transfer is preferable [7,8,11].

Skin grafting is one of the most common methods used to treat large defects, but its use should be limited when there is a healthy wound bed. Another concern that should be considered is joint stiffness caused by the contracture or pigmentation of the grafted surface. Thus, flap reconstruction is preferred over skin grafting of the hand. Small defects can be covered by locoregional flaps, whereas more sizable flaps are necessary when reconstructing medium- to large-sized defects. The muscle flap is a reliable option to control the infected area and cover the defect. However, it is sometimes too bulky to establish a good hand contour and requires skin grafting, which can lead to fibrosis, scarring, or contracture that requires a secondary procedure [12]. The fascial flap is an alternative method that avoids the bulkiness associated with the muscle flap and can provide a well-vascularized bed and ideal surroundings for tendon gliding. However, performing the revision procedure underneath the fascia can be more difficult than underneath a transferred flap. The main drawbacks of facial flaps relate to limitations in terms of size and thickness [13].

There are a paucity of reconstructive methods for large defects of the hand dorsum. The radial forearm flap, either pedicled or free, has been used for hand reconstruction. However, the unacceptable level of morbidity when sacrificing a named artery to the hand is a major problem with this approach. Furthermore, scar contracture, tendon exposure, or unappealing cosmetic results from the skin grafting on the donor site are additional concerns that preclude the use of the radial forearm flap, particularly in female patients [3,14]. The posterior interosseous flap with reverse flow can provide appropriate coverage for dorsal hand reconstruction. However, this approach involves the cutting of the posterior interosseous artery. The main drawback is that the flap dimensions are limited, as the donor site can only be closed primarily if the flap is less than 3–4 cm wide. Therefore, this approach is not frequently indicated in the treatment of hand dorsal defects [15,16]. The arterialized venous flap is a thin, pliable, relatively long-pedicled flap. It is easy to elevate this flap rapidly with no need to sacrifice a major artery. Nevertheless, it undergoes severe edema and bullae causing de-epithelization and discoloration from the immediate postoperative period. The venous flap requires an elaborate flap elevation with precise vein manipulation based on the surgeon’s experience. There is still uncertainty about this technique due to the lack of knowledge about the flap’s survival [17,18,19].

The lateral arm flap has been frequently employed to reconstruct damage to limbs and defects obtained from head and neck cancer ablation. It features a thin and pliable fasciocutaneous flap that creates a fine contour of the skin surface [20]. Donor morbidity can be minimized when allowing the direct closure of the site. Because the flap involves a reliable vascular plexus, the skin paddle can be tailored according to the surgeon’s needs [21,22]. Although the short pedicle can pose limitations, the flap can be used for a diverse range of defects. For example, three patients were treated with lateral arm flaps, and successful reconstruction was achieved. To overcome the short pedicle, further dissection was carried out to the level of the deep brachial artery in the spiral groove, with the detachment of the head of the triceps. Arterial anastomosis was performed on the dorsal branch of the radial artery. The pedicle was long enough to reach the recipient site on most occasions. The caliber of the vessels was sizable to establish end-to-side anastomosis. In the case of a sensate flap, the septal branch of the posterior antebrachial cutaneous nerve was isolated, harvested, and incorporated into the flap [23]. The donor site was less than 8 cm in width and was closed primarily, and the scar was less noticeable than those of other flaps.

Many studies have reported that newly developed perforator flaps can meet the needs of reconstruction with minimal morbidity [24,25]. The perforator is branched from the named major artery, entering the flap and supplying blood. The vessel diameter is 5 mm or less. Thus, it does not depend on a deep fascia layer or muscles to nourish the skin paddle [25,26]. The ALT flap is a fasciocutaneous flap nourished by one or several perforators pierced from the muscle. This flap has become a kind of workhorse flap for many surgeons because of its easy access, reliable anatomy, sizable dimensions, and sufficiently long pedicles. It is also one of the primary flaps used to treat a variety of defects on the hand [27,28]. The flap can not only be harvested together with muscle components, such as a chimeric flap, but also be tailored to split the skin paddle since it has multiple perforators. Moreover, the donor site is distant from the upper limb, so a two-team approach can be used to reduce the operative time. In hand reconstruction, there is no need to re-position the patient. The pedicle length and caliber are approximately 10 cm and 2 mm, respectively, meaning this approach can be reliably indicated in most patients requiring large coverage [27,29]. Generally, in order to resurface the hand, the flap needs to be thin and pliable. However, the flap sometimes needs to be thinned depending on the patient; thus, the flap should be raised on the supra-fascial plane in the middle of the fat layer to achieve the desired thickness. Here, there is a risk of disruption for optimal perfusion to the skin, leading to partial necrosis [30,31]. Due to this disadvantage, post-operative secondary procedures for thinning or debulking can be necessary, particularly in obese patients.

With the evolution of perforator-based microsurgery, the free superficial circumflex iliac artery perforator (SCIP) flap has been gaining popularity in autologous tissue reconstruction. The notable advantages, such as the easy flap design, relatively constant anatomy, and minimized morbidity of the donor site, enable surgeons to use this flap to treat various skin and soft tissue defects [5,32]. In particular, the thin and pliable nature of this flap makes it esthetically pleasing and results in acceptable motion in the hand [33,34]. Conversely, the flap has a relatively short and tiny pedicle and unpredictable vascular variation. Therefore, pre-operative imaging work-up and vascular mapping with ultrasound are critical for the success of the procedure.

This study is limited by the retrospective nature of the study. We believe that the prospective or randomized studies with further detailed assessment and large cohort should be continued.

## 5. Conclusions

The flap reconstruction of the hand dorsum is an excellent option providing satisfactory soft tissue coverage. Various flaps can be chosen according to the circumstances, but a thin, pliable flap of sufficient pedicle length is preferred. The use of the workhorse flap has shifted toward the perforator-based flap in order to minimize donor site morbidity. With this development, dorsal hand reconstruction outcomes are more promising.

## Figures and Tables

**Table 1:** Patients’ demographics.

Patient	Age(yr)	Sex	Injury	Area	Comorbidities	Flap	Flap Size (cm^2^)	Q-DASH	Esthetic Sartisfaction	Complications
1	55	F	Traffic injury	Right hand dorsum		SCIP *	9 × 7	2.5	Good	None
2	72	M	Traffic injury	Left hand dorsum	Metacarpal fractures	Lateral arm	10 × 6	2.6	Good	None
3	43	M	Crush injury	Right hand dorsum		Lateral arm	8 × 6	3.1	Fair	Partial necrosis
4	46	M	Crush injury	Left hand dorsum	Extensor tendon rupture	SCIP	11 × 8	2.6	Good	None
5	65	M	Traffic injury	Right hand dorsum		ALT ^¥^	13 × 7	1.5	Excellent	None
6	57	F	Traffic injury	Left hand dorsum	Wrist skin defect Extensor tendon rupture	ALT	12 × 8	4.2	Fair	None
7	32	M	Press injury	Left hand dorsum		Lateral arm	6 × 5	3.2	Good	None
8	34	M	Traffic injury	Right hand dorsum	Metacarpal fractures	ALT	12 × 7	2.1	Excellent	None
9	28	F	Crush injury	Right hand dorsum		SCIP	11 × 6	0	Excellent	None
10	65	F	Traffic injury	Left hand dorsum	Arterial rupture	SCIP	20 × 9	3.7	Fair	Joint stiffness
11	53	F	Traffic injury	Right hand dorsum		ALT	10 × 7	2.4	Good	None

* SCIP, superficial circumflex iliac artery perforator; ^¥^ ALT, anterolateral thigh.

## Data Availability

Data are contained within the article.

## Abbreviations

The following abbreviations are used in this manuscript:

ALT	Anterolateral thigh
SCIP	Superficial circumflex iliac artery perforator
